# Exercise‐induced changes in intramuscular total creatine concentration measured with ^1^H magnetic resonance spectroscopy: A pilot study

**DOI:** 10.14814/phy2.16171

**Published:** 2024-08-02

**Authors:** Xi Lin, Jiaying Zhang, Xiangwei Kong, Yanbin Li, Xueqin Xu, Lianjun Du, Jeff L. Zhang

**Affiliations:** ^1^ School of Biomedical Engineering ShanghaiTech University Shanghai China; ^2^ Central Research Institute, Shanghai United Imaging Healthcare Co., Ltd. Shanghai China; ^3^ Department of Radiology, Ruijin Hospital Shanghai Jiao Tong University School of Medicine Shanghai China

**Keywords:** calf muscles, magnetic resonance spectroscopy, plantar flexion, total creatine

## Abstract

Total amount of creatine (Cr) and phosphocreatine, or total creatine (tCr), may have a significant impact on the performance of skeletal muscles. In sports such as bodybuilding, it is popular to take Cr supplements to maintain tCr level. However, no study has explored the quantitative relationship between exercise intensity and the induced change in muscle's tCr. In this well‐controlled study, straight‐leg plantar flexion with specific load and duration was performed by 10 healthy subjects inside an MRI scanner, immediately followed by ^1^H MR spectroscopy (MRS) for measuring tCr concentration in gastrocnemius. For repeatability assessment, the experiment was repeated for each subject on two different days. Across all the subjects, baseline tCr was 46.6 ± 2.4 mM, ranging from 40.6 to 50.1 mM; with exercise, tCr significantly decreased by 10.9% ± 1.0% with 6‐lb load and 21.0% ± 1.3% with 12‐lb load (*p* < 0.0001). Between two different days, baseline tCr, percentage decrease induced by exercise with a 6‐lb and 12‐lb load differed by 2.2% ± 2.3%, 11.7% ± 6.0% and 4.9% ± 3.2%, respectively. In conclusion, the proposed protocol of controlled exercise stimulation and MRS acquisition can reproducibly monitor tCr level and its exercise‐induced change in skeletal muscles. The measured tCr level is sensitive to exercise intensity, so can be used to quantitatively assess muscle performance or fatigue.

## Graphical abstract

Using a well‐controlled exercise protocol and 1H MR spectroscopy, we observed that tCr levels decreased by about 10% with a 6‐lb load and about 20% with a 12‐lb load in the gastrocnemius. This linear relationship was repeatably observed on different days, indicating that tCr measurement by the proposed protocol can serve as a reliable quantitative marker for assessing muscle performance or fatigue.

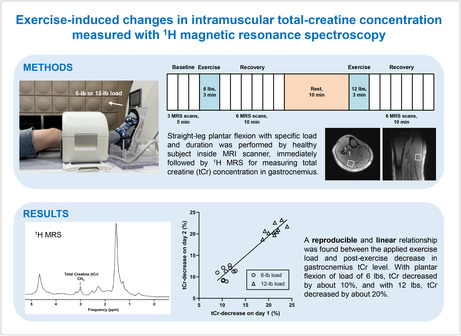

## 1 INTRODUCTION

Creatine (Cr) and its phosphorylated form (or phosphocreatine, PCr) play significant roles in the energy metabolism of skeletal muscles (Clark, 1997). The energy needed for muscle contractions is provided by the hydrolysis of adenosine triphosphate (ATP) into adenosine diphosphate (ADP) (Hargreaves & Spriet, 2020). Under anaerobic conditions, replenishment of ATP is mostly achieved by PCr's donating its phosphate group to ADP. Therefore, the amount of PCr and Cr available in skeletal muscles directly impacts the efficiency of the energy‐release process during exercise, and thus performance of the muscles. In sports, it is common for athletes and bodybuilders to supplement Cr as an ergogenic agent to improve performance (Izquierdo et al., 2002; Kreis, Kamber, et al., 1999). Creatine supplements are also taken by patients suffering from gyrate atrophy, muscular dystrophy, or neurodegenerative diseases (Brosnan & Brosnan, 2007). In addition, numerous studies observed delayed recovery of muscle PCr after exercise in patients with diseases such as diabetes mellitus, peripheral arterial disease, and cardiac failure, suggesting the potential of exercise‐induced PCr change in reflecting muscle‐mitochondrial impairment in the diseases (Crowther et al., 2003; Isbell et al., 2006; Taivassalo et al., 2001). Precise quantification of intramuscular Cr level before and after exercise would enable trainers to optimize their exercise routine or Cr supplementation regimen, as well as for precise assessment of patient's physical performance in the progression of related diseases.

In vivo measurement of Cr, PCr, or total creatine (tCr) in skeletal muscles can be achieved through MR spectroscopy (MRS) or imaging. For example, with a specialized radio‐frequency coil and pulse sequence, ^31^P MRS is widely used in measuring changes of PCr level during exercise or recovery of skeletal muscles (Khegai et al., 2018; Moll et al., 2018; Wcisło et al., 2014; Yoshida et al., 2013). Chemical Exchange Saturation Transfer Imaging (CEST) (Kogan, Haris, Debrosse, et al., 2014; Kogan, Haris, Singh, et al., 2014) has been shown to be promising in monitoring intramuscular Cr levels during and after exercise, particularly when exercise is mild enough to maintain a relatively stable level of muscle pH (McMahon et al., 2017). To measure tCr, one established noninvasive method is ^1^H MRS (Krssak et al., 2021). However, inconsistent results were reported regarding whether the measured tCr would decrease with exercise or not (Boesch & Kreis, 2001; Constantin‐Teodosiu et al., 1997; Kreis, Jung, et al., 1999; Rico‐Sanz et al., 1998). For example, tCr concentration in calf muscles of healthy subjects was measured to be in a wide range of 35 to 55 mM (Bottomley et al., 1997; Hwang et al., 2001; Kemp et al., 2007; Rico‐Sanz et al., 1998, 1999). The reported inconsistent results were presumably attributed to multiple factors, including variations in the used exercise protocol, MR sequences and parameters, and the specific muscles observed.

In this study, we tested the sensitivity and repeatability of an established ^1^H MRS method in measuring intramuscular tCr. Well‐controlled exercise stimulation was applied by having healthy subjects perform straight‐leg plantar flexion with specific load and duration inside an MRI scanner, and MRS was performed immediately before and after the exercise. The experiment was repeated in a same exam with exercise of different loads for sensitivity test, and was repeated on different days for the repeatability test. By this study, we aim to explore how intramuscular tCr changes with exercise and further whether such change can be reliably measured by the proposed approach.

## 2 METHODS

**FIGURE 1:**
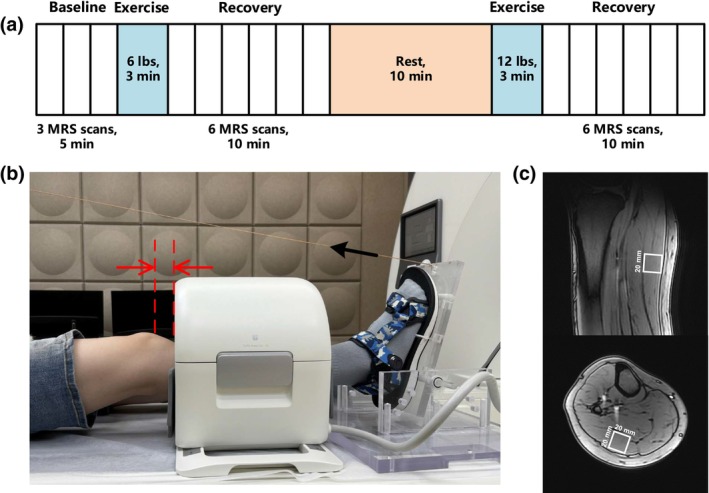
Experimental design for plantar‐flexion‐stimulated ^1^H MRS scan. (a) Timeline for the experimental procedures, including 2 exercise stimulations with load of 6 and 12 lbs, respectively. An extra 10‐min rest before the 12‐lb exercise was provided to avoid fatigue from the previous exercise. (b) Setup of subject's leg inside MRI scanner. A 24‐channel knee coil was used for scanning the right calf. The foot was attached to an MR‐compatible apparatus for loaded plantar flexion. For consistency, the distance between the apex of the subject's patella and the upper edge of the coil (red arrows) was kept the same for the 2 days' scans of each subject. The black arrow shows the inelastic rope that connects the apparatus to the load on the other end of the bed. (c) Positioning of the volume of interest (VOI) for MRS measurement in gastrocnemius muscle, viewed in sagittal and axial slices.

This study was approved by the local IRB committee. Ten healthy subjects (5 males and 5 females, age 23 ± 3 years, BMI 22.0 ± 3.3 kg/m^2^) were recruited with signed written consent in accordance with the Declaration of Helsinki. Before the experiment, subjects were asked to avoid strenuous exercise for 24 h, and not to drink alcohol or caffeinated drinks for 6 h. On two separate days (with a gap of 1.6 ± 1.1 days), the experiment protocol as shown in Figure 1a was repeatedly performed for each subject. In each experiment, MRS was measured before and after exercise stimulation of two different loads, using an MR‐compatible plantar flexion apparatus (Figure 1b). In the following, we introduce the details of the MRS acquisition and data analysis.

### 2.1 Exercise‐stimulated MRS acquisition

In a 3 T MR scanner (uMR890, United Imaging Healthcare, Shanghai, China), the subject was in a supine and foot‐first position, with the right calf covered by a 24‐channel knee coil. The right foot was fastened to the plantar‐flexion apparatus (Figure 1b). Setup of the leg was done to avoid any possible claudication of peripheral blood vessels. To ensure reproducible image acquisition for each subject, we measured the distance from the apex of the right patella to the upper edge of the coil, as shown by the red arrows in Figure 1b, and kept it the same for the 2 days' scans for the subject. On the head‐side of the bed, load was applied and attached to the foot‐side apparatus using an inelastic rope. Subjects first performed plantar flexion with a load of 6 lbs, with frequency of 1 Hz for 3 min, followed by a 20‐min break. Based on previous studies using a similar protocol (Wang et al., 2024; Zhang et al., 2019) and our preliminary experiment, a break of 20 min after the exercise of 6 lbs would be adequate for calf muscles to recover to its baseline state. To ensure proper execution of plantar flexion, we used a metronome to help the subject to perform the exercise with the desired frequency, and a team member was present by the scanner to supervise the exercise. Before and after the exercises, MRS acquisition as described below was performed.

For localization purpose, T_1_‐weighted gradient recalled echo images were acquired along three orientations, using the following parameter values: repetition time (TR) 11 ms, echo time (TE) 3.4 ms, flip angle 20°, bandwidth 290 Hz, FOV 300 × 300 mm^2^, matrix 256 × 256, slice thickness 6 mm. In the acquired images, a cuboid volume of interest (VOI) was positioned within the gastrocnemius of the subject. For consistency between different subjects, we fixed the volume of the VOI to be 8000 mm^3^, but adjusted its edges to ensure that the VOI was fully inside the gastrocnemius and avoided any subcutaneous fat or muscular fasciae (Figure 1c). For single‐voxel ^1^H MRS acquisition, a point‐resolved spectroscopy sequence (PRESS) with water suppression was performed with the following parameter values: TR 2000 ms, TE 30 ms, readout spectral width 1000 Hz, number of complex data points 1024, average times 48. Each MRS acquisition lasted about 1.5 min. At baseline, 3 MRS acquisitions were collected, and after each exercise stimulation, 6 acquisitions were done (Figure 1a). As a reference for frequency shift and an internal standard for concentration conversion, the same MRS protocol without water suppression was implemented with 5 average times before any exercise.

### 2.2 Quantification of tCr concentration from ^1^H MRS signals

The acquired spectrum data were processed using a Java‐based MR User Interface (jMRUI) (Naressi et al., 2001). Using the fitting routine AMARES (Vanhamme et al., 1997), the methyl protons of tCr were identified by fitting a Gaussian function to around 3.03 ppm of the water‐suppressed spectrum (De Graaf, 2019), and the area under the fitted curve (*A*_*c*_) after background correction was regarded as the amplitude of tCr signal. Fitting the water‐unsuppressed spectrum at around 4.7 ppm using Gaussian function, we obtained the area under the fitted curve (*A*_*w*_) as the water signal's amplitude. Based on the fitted results of *A*_*c*_ and *A*_*w*_, we calculated tCr concentration [tCr] using the following equation:

(1) $$\left[\mathrm{tCr}\right]=\frac{A_{c}}{A_{w}}\frac{N_{p,w}}{N_{p,c}}\frac{\left(1-e^{-\mathrm{TR}/T1,w}\right)e^{-\mathrm{TE}/T2,w}}{\left(1-e^{-\mathrm{TR}/T1,c}\right)e^{-\mathrm{TE}/T2,c}}C_{w}$$

where *N*_*p,c*_ of 3 and *N*_*p,w*_ of 2 are the number of protons contributing to the resonance in tCr and in water, respectively, and *C*_*w*_ as the concentration of water was assumed to equal 42.22 mol/L based on mean water content in adult muscle of 76% (Mingrone et al., 2001). Saturation effects of metabolites were corrected using reported values of T_1_ and T_2_ measured for human calf muscles (Krssak et al., 2004), with water T_1_ 1380 ms, water T_2_ 30 ms, tCr T_1_ 1000 ms and tCr T_2_ 135 ms.

### 2.3 Statistical analysis

To assess the sensitivity of tCr to exercise stimulation, we averaged the three estimates of tCr concentration measured before any exercise as the baseline, and averaged the six estimates after each exercise (with the load of 6 lbs or 12 lbs) as the postexercise level. Paired *t*‐test was used to evaluate whether across the 10 subjects, each of the two postexercise levels was significantly different from the baseline. A *p* value of less than 0.05 was regarded as significant. The percentage difference between the baseline and each of the postexercise levels was computed. The above analysis was separately conducted for the data acquired on two separate days.

To assess the repeatability of the measurements, we computed the differences between the two baseline tCr estimates measured on two separate days, the percentage change induced by 6‐lb exercise, and the percentage change induced by 12‐lb exercise, respectively. The inter‐day differences of each of these parameters for all 10 subjects were averaged. For demonstration, the postexercise changes of the different days were also compared using a Pearson correlation plot to assess the correlation degree and potential bias, and using Bland–Altman plot to display the magnitude and the trend of difference across the range of measurement (Bland & Altman, 1986). All statistical analyses were performed in the environment of GraphPad Prism (version 10.1.2; GraphPad Software, La Jolla, CA, USA).

## 3 RESULTS

**FIGURE 2:**
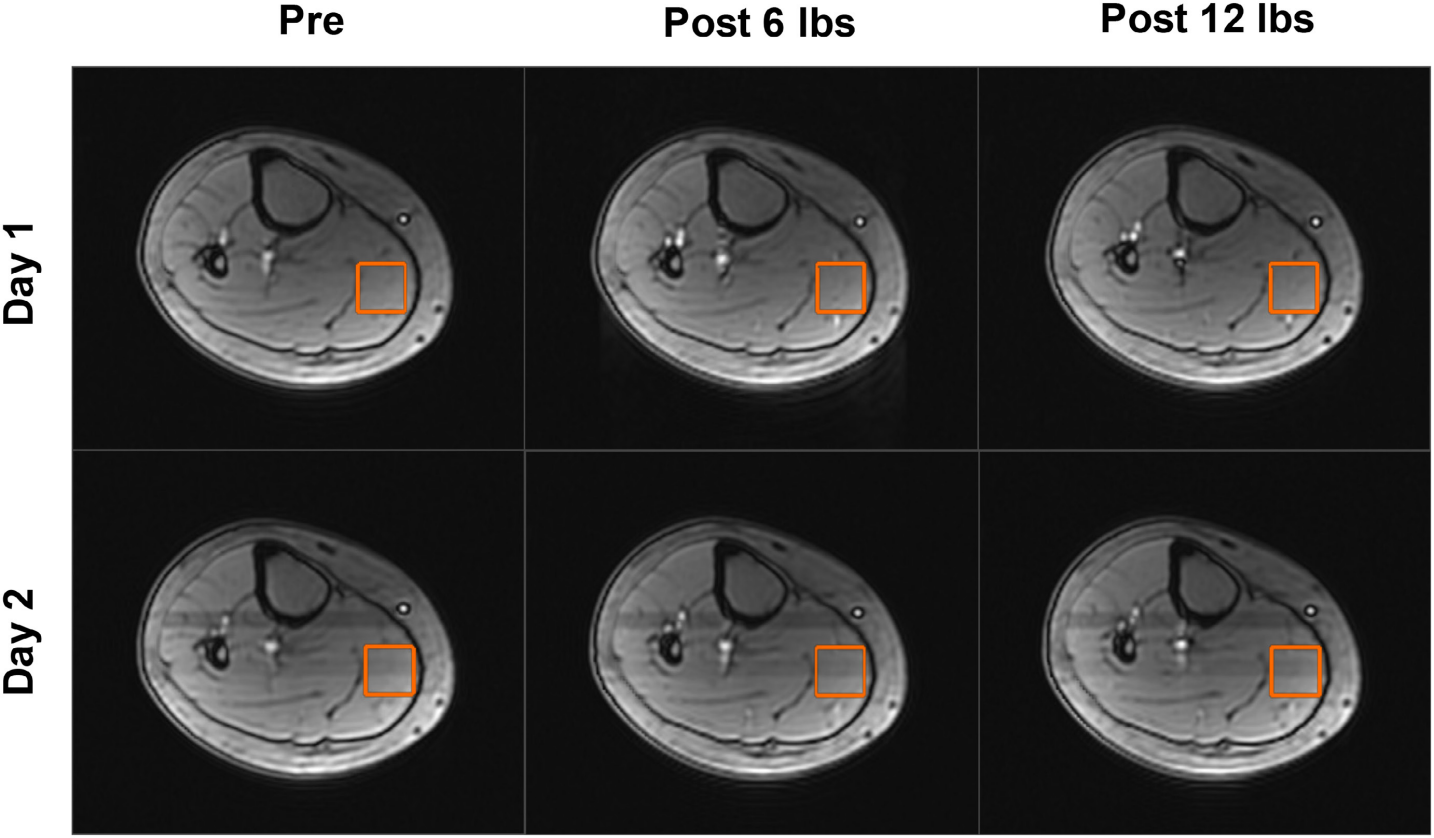
Example of positioning MRS volume of interest (VOI) for one subject, viewed in axial slices. VOIs for MRS acquisitions before (pre) and after (post) exercise stimulation on two separate days were quite similar, suggesting the high consistency of our VOI‐positioning approach.

**FIGURE 3:**
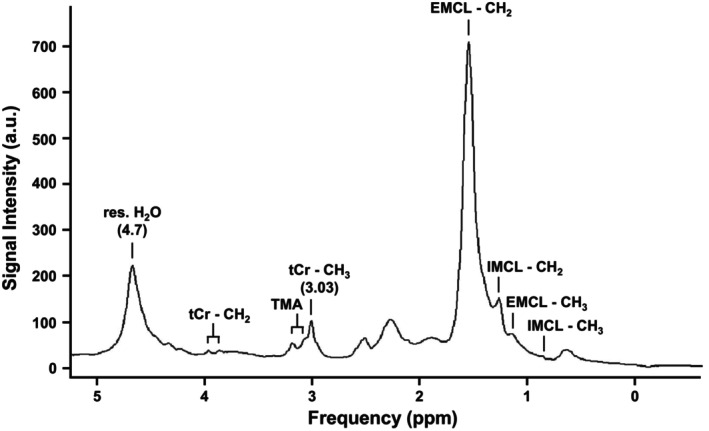
Example of a water‐suppressed ^1^H MR spectrum measured from the gastrocnemius muscle of a 23‐year‐old healthy male (PRESS, TE/TR = 30/2000 ms, 48 averages). The signal from the methyl group of total creatine is located at 3.03 ppm. Other peaks originated from trimethylamine complex (TMA), intramyocellular lipids (IMCL), extramyocellular lipids (EMCL), and residual water.

To precisely monitor [tCr] with MRS on different time points, it is important to consistently position the volume for generating MRS signals. Figure 2 shows for one subject the positioning of MRS volume of interest before and after exercise on two separate days, indicating that using our positioning approach, the VOI for MRS acquisition can be positioned consistently. A representative example of ^1^H MR spectrum acquired in our study is shown in Figure 3. In this example, the peak at 3.03 ppm that corresponds to the methyl group of total creatine was visually detectable and fitted by a Gaussian function for further quantification.

**FIGURE 4:**
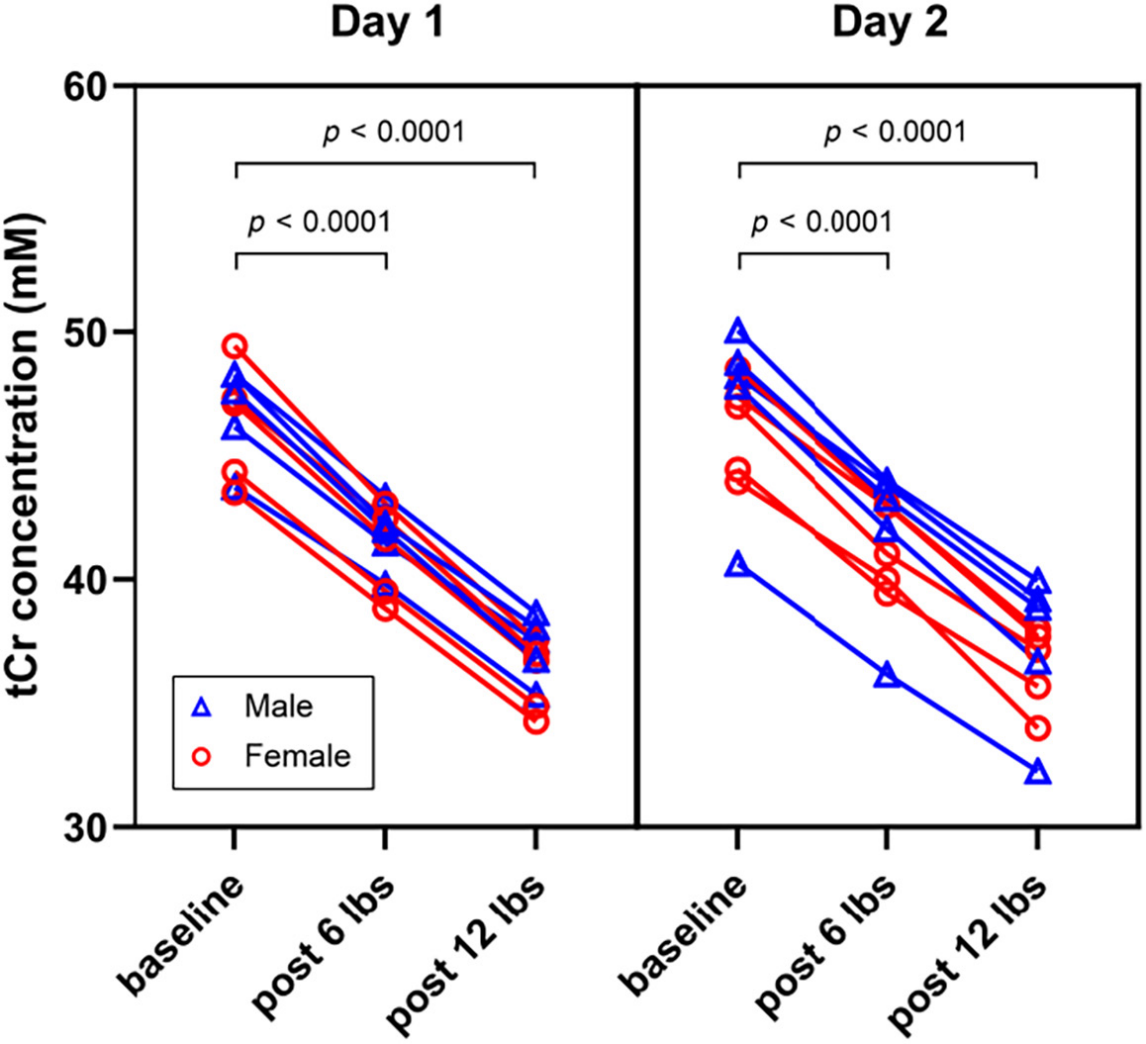
The measured concentration of total creatine at baseline, after exercise with 6‐lb load, and after exercise with 12‐lb load on two separate days. Data are displayed separately for males (blue triangles) and females (red circles). Across the 10 subjects, both the two postexercise levels were significantly different from the baseline (*p* < 0.0001).

In Table 1, we list the baseline estimates of [tCr] measured in gastrocnemius of 10 healthy subjects on two separate days, along with their respective percentage decrease after exercise stimulation with load of 6 and 12 lbs. On day one, baseline [tCr] for the 10 subjects was 46.6 ± 2.1 mM, with a range from 43.5 to 49.4 mM. After exercise with load of 6 lbs and 12 lbs, [tCr] decreased by 11.0% ± 1.2% and 21.2% ± 1.6%, respectively (*p* < 0.0001). On day two, the baseline [tCr] was 46.7 ± 2.8 mM, with a range from 40.6 to 50.1 mM, and decreased by 10.9% ± 1.3% and 20.8% ± 1.3% after exercise with load of 6 lbs and 12 lbs respectively. It is noted that variation among the baseline levels of the different subjects as quantified by the coefficient of variation was less than 7%, and that the exercise‐induced decreases in [tCr] were approximately proportional to the applied loads. The results are also displayed in Figure 4.

**TABLE 1:** Measurements of tCr concentration (mM) at baseline and after exercise with load of 6 lbs and 12 lbs, on two separate days. Ten healthy subjects were recruited for the study. Mean and SD of the measurements for the 10 subjects were computed.

Subject	Day 1			Day 2		
	Baseline (mM)	Post 6 lbs (%)	Post 12 lbs (%)	Baseline (mM)	Post 6 lbs (%)	Post 12 lbs (%)
1 (m)	46.1	10.2%	18.7%	48.2	9.1%	18.7%
2 (m)	43.7	9.0%	19.2%	40.6	11.0%	20.6%
3 (f)	44.4	10.9%	21.3%	43.9	8.9%	22.6%
4 (m)	48.3	10.2%	20.0%	50.1	12.1%	20.2%
5 (f)	47.3	10.2%	21.8%	47.4	9.3%	20.4%
6 (f)	49.4	12.9%	24.0%	48.5	11.4%	21.7%
7 (f)	47.1	11.6%	22.1%	47.0	12.7%	20.9%
8 (f)	43.5	10.8%	21.3%	44.4	11.3%	19.7%
9 (m)	48.3	12.5%	21.1%	48.7	11.2%	20.3%
10 (m)	47.6	11.8%	22.8%	47.8	12.1%	23.2%
Average	46.6 ± 2.1	11.0% ± 1.2%	21.2% ± 1.6%	46.7 ± 2.8	10.9% ± 1.3%	20.8% ± 1.3%

When comparing the results of the two separate days, the baseline [tCr] differed by 1.0 ± 1.0 mM or 2.2% ± 2.3%; the decrease of [tCr] induced by exercise with load of 6 lbs differed by 1.3% ± 0.6% (11.0% ± 1.2% on day 1 vs 10.9% ± 1.3% on day 2), or a relative difference of 11.7% ± 6.0%; [tCr] decrease induced by exercise of 12 lbs differed by 1.1% ± 0.7% (21.2% ± 1.6% on day 1 vs 20.8% ± 1.3% on day 2), or a relative difference of 4.9% ± 3.2%. These results suggest that in this well‐controlled setting, both the baseline and the exercise‐induced change were measured with high repeatability.

**FIGURE 5:**
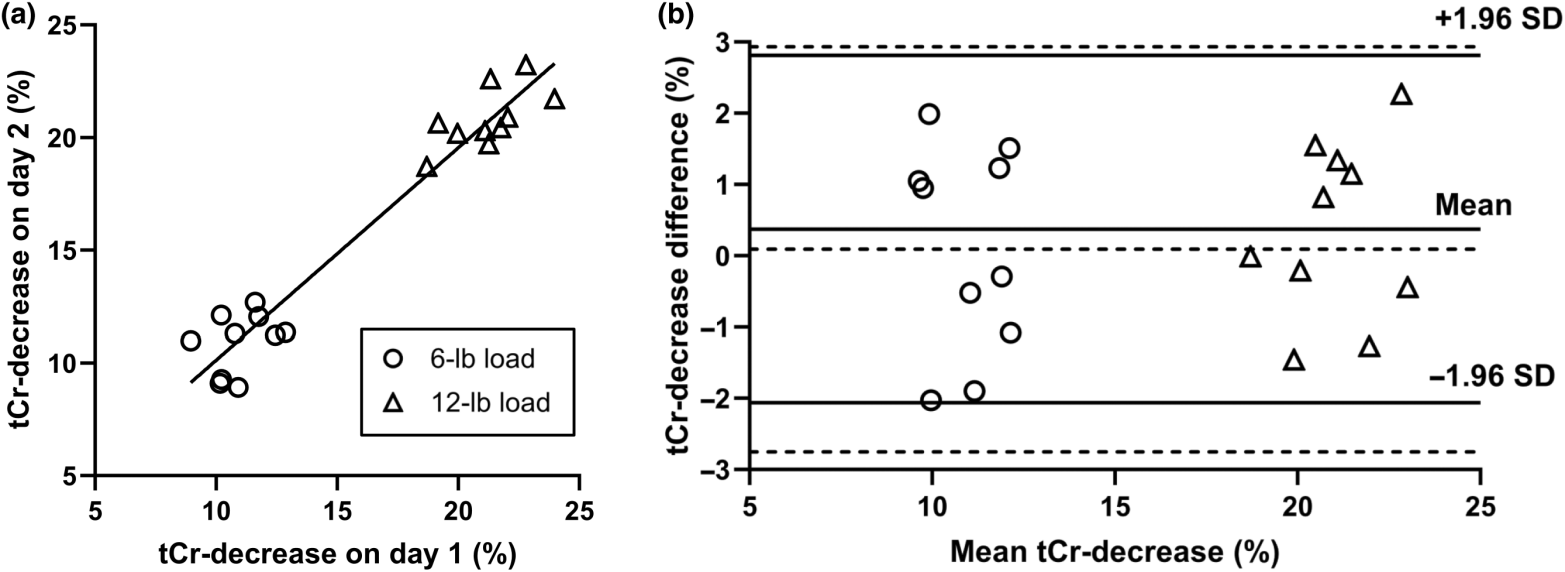
Comparison analysis of exercise‐induced changes in tCr concentration measured on two separate days in gastrocnemius of healthy subjects. (a) Pearson correlation plot of the two groups of estimates. Circle and triangle symbols represent decreases of tCr level after exercise with load of 6 lbs and 12 lbs, respectively. The correlation coefficient was 0.97 (*p* < 0.0001), and the equation for the linear regression was D2 = 0.94 × D1 + 0.71. (b) Bland–Altman plot for the two groups of estimates showed similar 95% limits of agreement: With mean difference ± 1.96 SD at −2.75% and 2.93% (dashed lines) for exercise with load of 6 lbs, and − 2.06% and 2.81% (solid lines) for 12 lbs.

The correlation plot for the exercise‐induced decreases in [tCr] measured on two separate days is shown in Figure 5a. Between the 2 days' estimates, the correlation coefficient was 0.97 (*p* < 0.0001), and the regression line was D2 = 0.94 × D1 + 0.71, suggesting high repeatability for the measured exercise‐induced change in [tCr]. Bland–Altman plot of the repeated measurements is shown in Figure 5b. For the percentage decreases after exercise with load of 6 lbs, the 95% limits of agreement were −2.75% and 2.93%, and for 12 lbs the limits were −2.06% and 2.81%. The limits were similar for the two different loads, showing a consistent level of estimation error across the range of measurement.

## 4 DISCUSSION

With the proposed approach, we measured total Cr concentration in gastrocnemius of a group of young healthy subjects to be in the range of about 40–50 mM, which was consistent with previous measurements using biopsy or other MRS methods (Bottomley et al., 1997; Kemp et al., 2007; Rico‐Sanz et al., 1999). With well‐controlled stimulations of plantar flexion, we observed a consistent decrease of about 10% in tCr level for exercise load of 6 lbs, and a decrease of about 20% for exercise load of 12 lbs, suggesting a linear relationship between the applied exercise load and the induced decrease in total Cr concentration. With proper control of the subjects' physical activity, the measurements on separate days were quite comparable.

The measured decreases in total Cr concentration after exercise can be attributed to the increased usage of muscles and therefore higher generation rate of creatinine. In muscle metabolism, both Cr and phosphocreatine spontaneously and nonenzymatically convert to creatinine (Brosnan & Brosnan, 2007), some of which diffuses out of muscle cells and is excreted from the kidneys as urine. This is the basis for the clinical approach of using clearance of serum creatinine for estimating renal function. During exercise of higher intensity, there is an increase in muscle breakdown and turnover, leading to acute increases in creatinine levels (Beetham et al., 2018). The loss of tCr (as creatinine) is either restored by endogenous synthesis in the kidneys, pancreas and liver (Nasrallah et al., 2010), or by dietary intake mostly from meat and dairy products. Our measurements were performed immediately after the exercise stopped, so the measured change in tCr level reflects subject's exercise performance or fatigue, which is also supported by the linearity between the applied exercise load and the measured [tCr] decrease. Compared to other methods for assessing muscle performance, the measured tCr level by the proposed method is derived from an individual muscle group.

Inter‐day repeatability is usually difficult to assess for most physiologic measurements, as strictly controlling a subject's physiologic state is hard. In this study, instead of assessing the absolute concentration of tCr in the muscle, we focused on their changes following exercise stimulation. Such exercise‐induced change in activated muscles primarily reflects muscle's workload, and is less affected by other factors than the baseline level is. In our preliminary study with few cases, we concluded that for repeatable MRS results, localization of the volume of interest for generating MR spectrum needs to be quite consistent. For this reason, for the different measurements of each subject, we fixed the distance from the apex of the subject's patella to the upper edge of the coil. The other factor reported to possibly affect MRS repeatability is the orientation of muscle fibers within the volume of interest relative to the B_0_ field (Krssak et al., 2021), which presumably complicates the spectral features. To avoid potential artifacts from this factor, we ensured in this study that the orientation of the VOI was consistent across the different measurements.

This study has multiple limitations. First, as a pilot study for testing the sensitivity of tCr level to exercise, we used low and moderate loads only to stimulate calf muscles, and the exercise duration was only 3 min. In future studies, stimulation of strenuous and/or intense exercise might be explored. Second, in this study we used young healthy subjects only, excluding either elderly or diseased subjects. The other factor we missed was skeletal muscles with different fiber types as gastrocnemius. Third, we did not investigate the potential difference between the two heads of gastrocnemius, but assumed that the two heads reacted similarly in the exercises. In future work, comparison of the two gastrocnemius heads and even other calf muscle groups will be carried out, to see how the different muscles would cooperate to perform the exercise.

## 5 CONCLUSION

In conclusion, based on an established MRS protocol, we managed to detect changes in tCr concentration after exercise in calf muscles. The measured changes in tCr were sensitive to exercise of low and medium loads and highly repeatable, suggesting its potential value for quantitative assessment of individual muscle's performance or fatigue.

## AUTHOR CONTRIBUTIONS

X.L. and J.L.Z. conceived and designed research; X.L., J.Z., X.K. and Y.L. performed experiments; X.L. analyzed data; X.L. interpreted results of experiments; X.L. and J.Z. prepared figures; X.L. drafted manuscript; X.L., J.Z., X.K. and J.L.Z. edited and revised manuscript; X.L., J.Z., X.K., Y.L., X.X., L.D., and J.L.Z. approved final version of manuscript.

## FUNDING INFORMATION

This work was supported by National Natural Science Foundation of China, Grant No.82171924 (to Jeff L. Zhang).

## CONFLICT OF INTEREST STATEMENT

The authors have no conflict of interest to declare.

## ETHICS STATEMENT

All experiments were approved by the Research Ethics Committee of ShanghaiTech University (ShanghaiTech BME IRB#2021–008).

## Data Availability

Data will be made available upon reasonable request from the corresponding author.
